# Serum metabolism characteristics of patients with myocardial injury after noncardiac surgery explored by the untargeted metabolomics approach

**DOI:** 10.1186/s12872-024-03736-y

**Published:** 2024-02-03

**Authors:** Yuanjia Zhang, Mengjia Kou, Kuanzhi Liu, Yaqing Zhan, Weiyi Xu, Chanyan Huang, Wenqi Huang, Xu Zhao

**Affiliations:** Department of Anesthesiology, The First Affiliated Hospital, Sun Yat-sen University, No. 58 Zhongshan Er Road, Guangzhou, China

**Keywords:** Myocardial injury after noncardiac surgery, Untargeted metabolomics, Metabolic pathway, Lipid metabolism

## Abstract

**Background:**

Myocardial injury after noncardiac surgery (MINS) is one of the most common complications associated with postoperative adverse cardiovascular outcomes and mortality. However, MINS often fails to be timely diagnosed due to the absence of clinical symptoms and limited diagnostic methods. The metabolomic analysis might be an efficient way to discover new biomarkers of MINS. Characterizing the metabolomic features of MINS patients may provide new insight into the diagnosis of MINS.

**Methods:**

In this study, serum samples from 20 matched patients with or without MINS (*n* = 10 per group) were subjected to untargeted metabolomics analysis to investigate comprehensive metabolic information. Differential metabolites were identified, and the enriched metabolic pathway was determined based on the Kyoto Encyclopedia of Genes and Genomes (KEGG) database.

**Results:**

A comprehensive analysis revealed 124 distinct metabolites, predominantly encompassing lipids, amino acids and other compounds. The observed modifications in metabolic pathways in patients with or without MINS showed significant clustering in cholesterol metabolism, aldosterone synthesis and secretion, primary bile acid biosynthesis, as well as cysteine and methionine metabolism. Four specific metabolites (taurocholic acid, L-pyroglutamic acid, taurochenodeoxycholic acid, and pyridoxamine) exhibited promising potential as biomarkers for prognosticating MINS.

**Conclusions:**

This study contributes valuable insights into the metabolomic features of MINS and the discovery of potential biomarkers which may help the early diagnosis of MINS. The identified metabolites and altered pathways offer valuable insights into the molecular underpinnings of MINS, paving the way for improved diagnostic approaches and potential intervention strategies.

**Supplementary Information:**

The online version contains supplementary material available at 10.1186/s12872-024-03736-y.

## Introduction

Myocardial injury after noncardiac surgery (MINS) is an independent prognosticator of postoperative mortality [1, 2], which occurs in 5–25% of adult patients within 30 days following noncardiac surgery [3, 4]. However, 90% of MINS patients were asymptomatic or presented with atypical symptoms [3, 5]. The postoperative analgesia and surgical stress likely covered distinct presentations of chest pain or other ischemic symptoms [6, 7]. At present, the diagnosis of MINS relies on routine postoperative troponin screening, where elevated troponin levels often indicate perioperative myocardial injuries [3, 5]. Yet, previous studies have shown that approximately 80% of patients with MINS did not receive a timely diagnosis for clinically asymptomatic symptoms [8]. Therefore, more sensitive and specific biomarkers to screen MINS patients are still needed [9].

Metabolomics, a newly developed investigative technique, has shown potential for the figuring of metabolic features and discovering new biomarkers for specific diseases [10]. This method allows for the analysis of small molecules present in biological fluids and tissues, thereby revealing metabolic profiles under both physiological and diseased states [11, 12]. In particular, untargeted metabolomics, characterized as a “nonspecific approach”, enables the comprehensive investigation of all metabolic substances, including both endogenous and exogenous compounds [13]. Therefore, examining metabolic markers during perioperative periods may provide valuable insights into perioperative complications. Prior studies have documented new metabolic biomarkers and potential pathological metabolic signals in various biological samples of patients who have experienced myocardial ischemia-reperfusion injury [14]. However, only a limited number of studies have provided a comprehensive metabolomic profile of MINS or a thorough examination of perioperative metabolite changes. Consequently, characterizing the metabolomic features of MINS patients may provide new insight into the diagnosis of MINS.

This study employed an untargeted metabolomic analysis to discern the metabolic disparities in serum from patients with or without MINS. The compounds identified through untargeted metabolomics serve as potential biomarkers of MINS. The ascertained metabolic alterations in patients with MINS may offer novel avenues for future exploratory investigations.

## Methods

### Study population

The study analyzed the serum samples obtained from a previous prospective study (ChiCTR2200055929, https://www.chictr.org.cn/showprojEN.html?proj=134260). Sixty-seven patients who underwent elective noncardiac surgery at the First Affiliated Hospital of Sun Yet-sun University between October 2021 and May 2022, were included in the previous study. Ethical approval was obtained from the Ethics Committee of the First Affiliated Hospital of Sun Yet-sun University (Guangzhou, China), and informed consent was obtained from the patients for utilizing frozen serums in subsequent analyses.

### Selection of MINS and non-MINS patients

Among the enrolled 67 patients, 10 were identified as having MINS, defined as the serum concentration of postoperative troponin T exceeding 0.03 ng·ml^− 1^ in the absence of nonischemic causes for troponin T elevation [15]. Another 10 patients without postoperative troponin T elevation were selected using the propensity score matching. Variables including age, sex, body mass index, preoperative hypertension or diabetes, and surgery type were used during the matching process. Thereafter, 10 pairs of MINS or non-MINS patients were selected.

### Blood sample collecting

All blood samples were collected post-surgery in the operating room or in the wards. After centrifugation at 3,000 g at 4 °C for 10 minutes, the resulting supernatant was extracted. The serum samples were stored at -80 °C and used for subsequent untargeted metabolomics.

### UHPLC-MS/MS analysis

Ultra-high-performance liquid chromatography-tandem mass spectrometry (UHPLC-MS/MS) analysis was conducted using a Vanquish UHPLC system (ThermoFisher, Germany) coupled with an Orbitrap Q Exactive TM HF mass spectrometer (Thermo Fisher, Germany) at Novogene Co., Ltd. (Beijing, China). The serum samples from MINS and non-MINS patients were separated using a Hypersil Gold column (2.1 × 100 mm, 1.9 μm) at a flow rate of 0.2 mL/min. The elution used 0.1% formic acid in water and methanol in the positive polarity mode, while 5 mM ammonium acetate (pH 9.0) and methanol in the negative polarity mode. The solvent gradient was a pattern of 2-100% B from 0 to 10 minutes, followed by 100-2% B from 10.1 to 12 minutes. The Q Exactive TM HF mass spectrometer was utilized in both polarity modes, with a spray voltage of 3.5 kV and a capillary temperature of 320 °C. The sheath gas flow rate was set at 35 psi, the aux gas flow rate was adjusted to 10 L/min, the S-lens RF level was set to 60, and the heater temperature was maintained at 350 °C. In order to conduct a comprehensive analysis of the initial data obtained from the Compound Discoverer 3.1 (CD3.1, ThermoFisher), peak alignment, peak picking, and quantitation were recorded. Subsequently, accurate qualitative and relative quantitative results were obtained by comparing them with the mzCloud, mzVault, and MassList databases, which were then subjected to statistical analysis.

### Statistics

Data quality control (QC) using Pearson correlation was performed before formal analysis to ensure accurate, reliable, and stable metabolic characteristics. Discriminative pattern analysis of the MINS and non-MINS patients was conducted using principal component analysis (PCA) and partial least squares discriminant analysis (PLS-DA). In order to be considered as differential metabolites, certain criteria had to be met, including variable importance in the projection (VIP) > 1 and fold change ≥ 2 or ≤ 0.5, as well as a univariate *p*-value (t-test) < 0.05. Volcano plots were employed with the purpose of screening metabolites, utilizing log2 (fold change) and -log10 (*p*-value) of metabolites, implemented through ggplot2 in the R software. For the creation of clustering heatmaps, the data underwent normalization via z-scores of the intensity areas of the differential metabolites, and were visualized using the pheatmap package in the R software. The Kyoto Encyclopedia of Genes and Genomes (KEGG) database (https://www.genome.jp/kegg/pa-thway.html) was utilized to evaluate the statistically enriched metabolic pathways of filtered metabolites. The *p*-value < 0.05 obtained through the hypergeometric test method was considered as the threshold of the KEGG significantly enriched pathways of potential importance. The receiver operating characteristic (ROC) curve and the area under the curve (AUC) value were obtained using the pROC package in the R software. For clinical characteristics, Fisher exact test and Mann-Whitney U test were used to examine the disparities in MINS or non-MINS patients.

The metaX software was employed to perform PCA and PLS-DA for metabolomics data analysis. The SPSS software (version 25.0, SPSS Inc., Chicago, IL) was employed to analyze the clinical characteristics data.

## Results

### Characteristics of MINS and non-MINS patients

Table 1 presents the characteristics of MINS and non-MINS patients. Age, sex, body mass index, comorbidities, surgery type, and preoperative hypertension-related medication were balanced between groups.

**Table 1:** Patient characteristics ^a^

	All (*n* = 20)	Non-MINS (*n* = 10)	MINS (*n* = 10)	*P*-value
**TnT concentration** **(ng·ml**^**− 1**^**)**	0.020 (0.005–0.042)	0.005 (0.004–0.008)	0.042 (0.033–0.067)	< 0.001
**Clinical characteristics**				
Age (year)	63 (53–71)	61 (53–68)	63 (54–75)	0.677
Male Sex (n, %)	11 (55%)	6 (60%)	5 (50%)	0.661
Female Sex (n, %)	9 (45%)	4 (40%)	5 (50%)	
BMI (kg·m^-2^)	22 (20–24)	24 (21–25)	22 (19–24)	0.241
ASA III (n, %)	20 (100%)	10 (100%)	10 (100%)	> 0.999
Epidural analgesia (n, %)	9 (45%)	3 (30%)	6 (60%)	0.370
Smoking history (n, %)	6 (30%)	2 (20%)	4 (40%)	0.629
**Surgery type (n, %)**				
Hepatobiliary	8 (40%)	2 (20%)	6 (60%)	0.189
Gastrointestinal	9 (45%)	6 (60%)	3 (30%)	
Lung	3 (15%)	2 (10%)	1 (10%)	
**Comorbidities (n, %)**				
Hypertension	10 (50%)	5 (50%)	5 (50%)	> 0.999
Coronary heart disease	1 (5%)	0 (0%)	1 (10%)	> 0.999
Peripheral vascular disease	2 (10%)	1 (10%)	1 (10%)	> 0.999
Diabetes	3 (15%)	2 (20%)	1 (10%)	> 0.999
Gastrointestinal disease	1 (5%)	0 (0%)	1 (10%)	> 0.999
Liver disease	5 (25%)	1 (10%)	4 (40%)	0.303
Kidney disease	2 (10%)	1 (10%)	1 (10%)	> 0.999
History of surgery	7 (35%)	4 (40%)	3 (30%)	> 0.999
**Preoperative medication (n, %)**				
ARB or ACEI	3 (15%)	1 (10%)	2 (20%)	> 0.999
Beta blocker	2 (10%)	1 (10%)	1 (10%)	> 0.999
Calcium channel blocker	5 (25%)	3 (30%)	2 (20%)	> 0.999

^a^ Variables are present as numbers (percentages) or median (IQR), as appropriate

ACEI, angiotensin converting enzyme inhibitor; ARB, angiotensin receptor blocker; ASA, American Society of Anesthesiology; BMI, body mass index; IQR, interquartile range; MINS, myocardial injury after noncardiac surgery; TnT, troponin T

### Metabolic characteristics detected in human serum samples using untargeted metabolomics

**Fig. 1:**
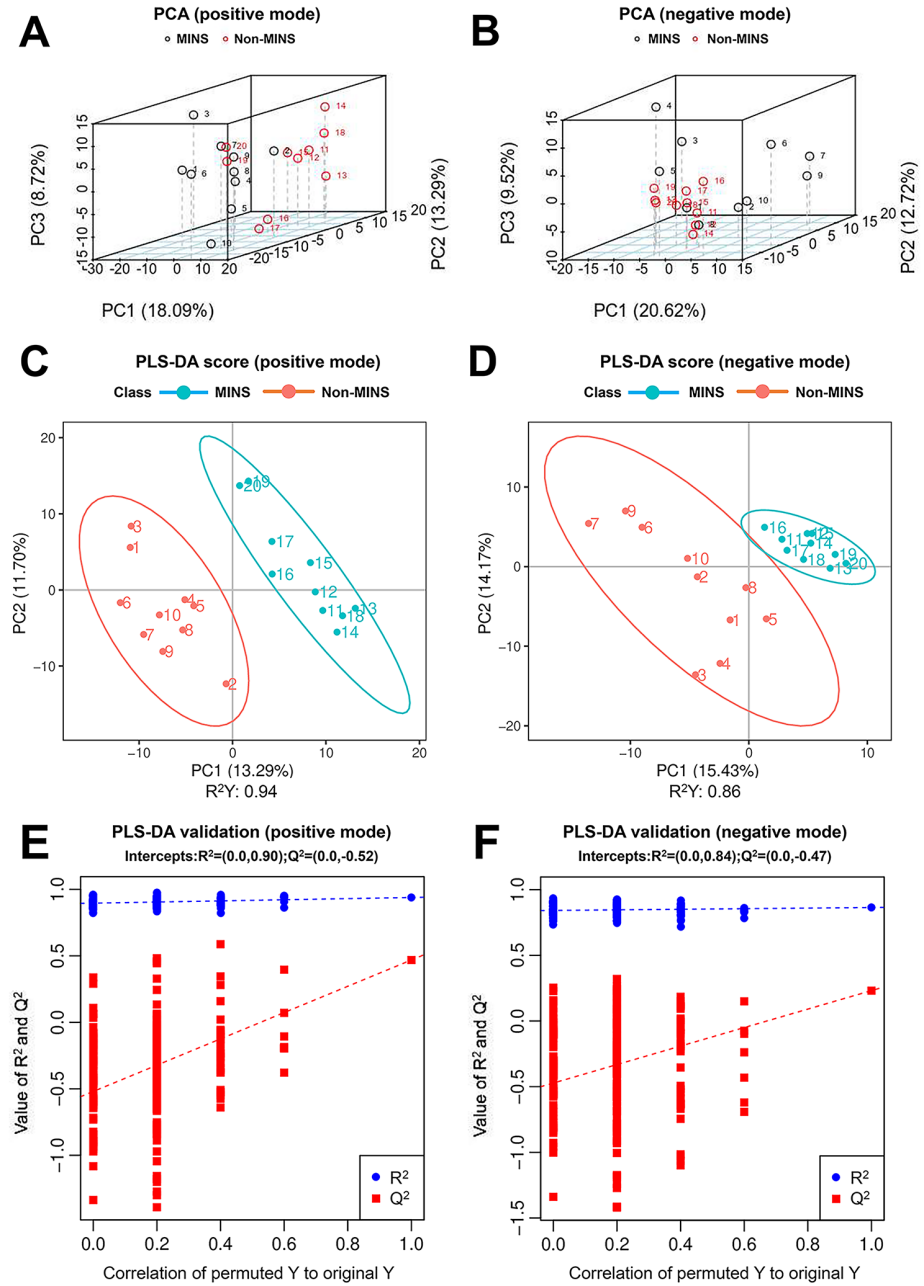
The metabolomic profile in MINS and non-MINS patients. (**A**) PCA in positive mode; (**B**) PCA in negative mode. Samples from MINS and non-MINS patients are shown in black and red, respectively. (**C**) PLS-DA in positive mode (R^2^Y: 0.94); (**D**) PLS-DA in negative mode (R^2^Y:0.86). Samples from MINS and non-MINS patients are shown in red and blue, respectively. The X and Y axes represent the individuals’ contributions to the first two principal components (PC1 and PC2). (**E**) Statistical validation of PLS-DA model in positive mode; (**F**) Statistical validation of PLS-DA model in negative mode. The intercepts of R^2^ = (0.0, 0.90) and Q^2^ = (0.0, − 0.52), R^2^ = (0.0, 0.84) and Q^2^ = (0.0, − 0.47), indicate that the PLS-DA model is not overfitted. MINS, myocardial injury after noncardiac surgery; PCA, principal component analysis; PLS-DA, partial least squares discrimination analysis

The chromatograms obtained in both positive and negative ion modes (Additional Figure S1A, B) exhibit satisfactory overlap. The Pearson correlation (Additional Figure S1C, D) of positive electrospray ionization (ESI+) and negative ESI (ESI-) QC sample demonstrated a high level of correlation, indicating a stable detection process and reliable data quality. In total, 945 metabolites were screened, with 631 detected at (ESI+) ion mode and 314 detected at (ESI-) ion mode. The PCA of serum samples (Fig. 1A, B) demonstrated a distinct distribution trend for distinguishing the MINS and non-MINS patients. The PLS-DA score plot (Fig. 1C, D) effectively separates the two groups of patients, achieving a cumulative R^2^Y of 0.94 at the positive ionization mode and 0.86 at the negative ionization mode. Furthermore, two hundred random disruption and subsequent modeling tests (Fig. 1E, F) revealed negative intercept values of Q^2^ statistic for both ion modes, indicating that the PLS-DA models were not overfitting.

**Fig. 2:**
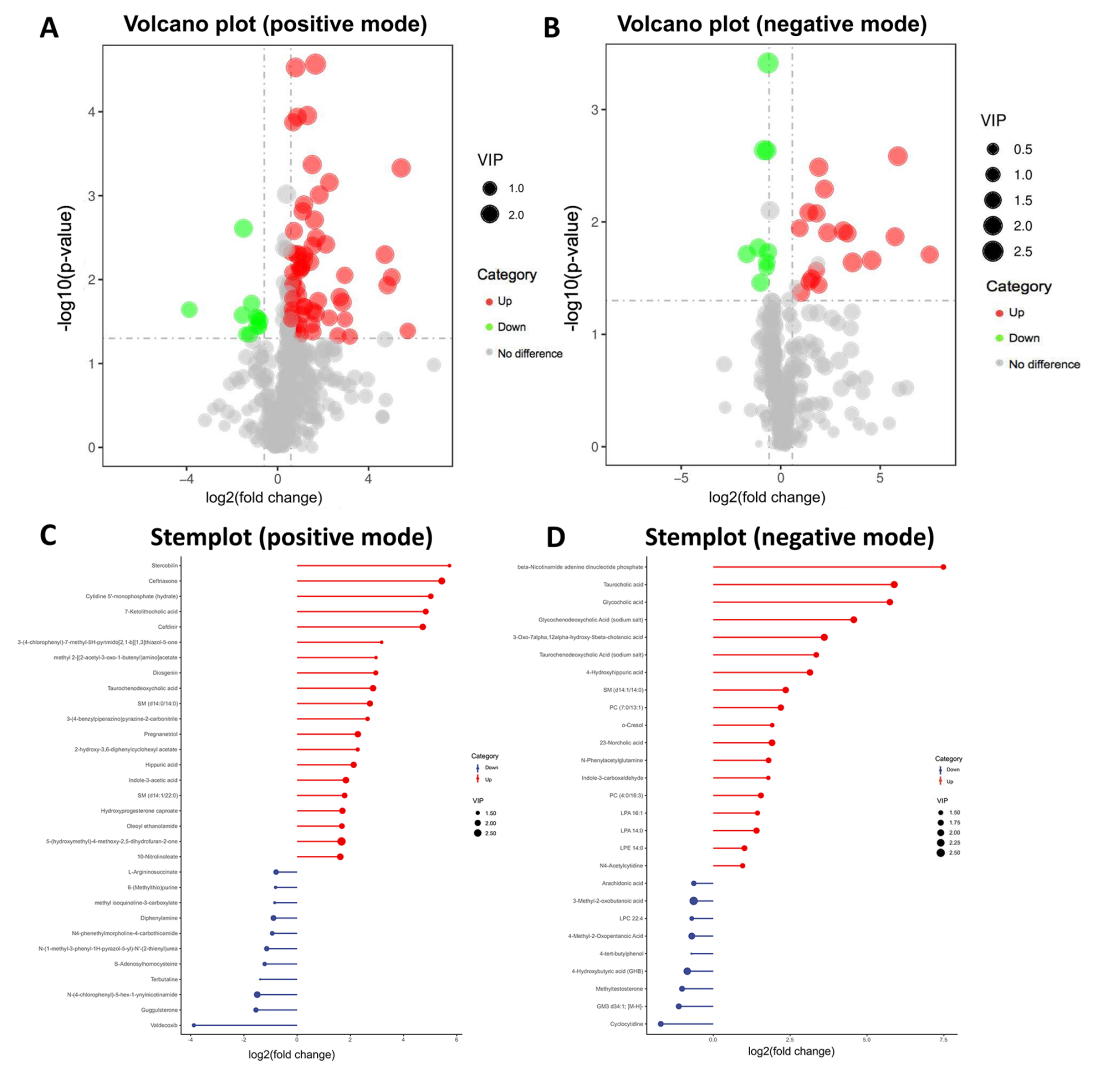
Screening of differential metabolites in MINS and non-MINS patients. (**A**) volcano plot in positive mode; (**B**) volcano plot in negative mode. The twofold abundance difference threshold is denoted by the vertical dashed lines, while the *p* = 0.05 threshold is indicated by the horizontal dashed line. Metabolites exhibiting significant changes are depicted in red (up-regulated) or green (down-regulated). (**C**) stemplot in positive mode; (**D**) stemplot in negative mode. Stemplots provide a clear depiction of the modification of the top 20 metabolites with substantial differences. The length of the stem in the plot corresponds to the magnitude of the log2 (fold change), while the size of the point represents the value of VIP. Metabolites exhibiting significant alterations are denoted by red dots (indicating upregulation) or blue dots (indicating downregulation). MINS, myocardial injury after noncardiac surgery; VIP, variable importance in the projection

Among the metabolites analyzed, 95 at positive mode (84 upregulated and 11 down-regulated) and 29 at negative mode (19 upregulated and 10 down-regulated) were significantly distinguishable between the MINS and non-MINS patients (Additional Table S1). The volcano plot (Fig. 2A, B) visually represents the distribution of these differential metabolites, with each metabolite represented by a colorful dot. Additionally, the stem plot (Fig. 2C, D) effectively indicates the direction of regulation (up or down) and highlights metabolites with large differences in expression levels.

**Fig. 3:**
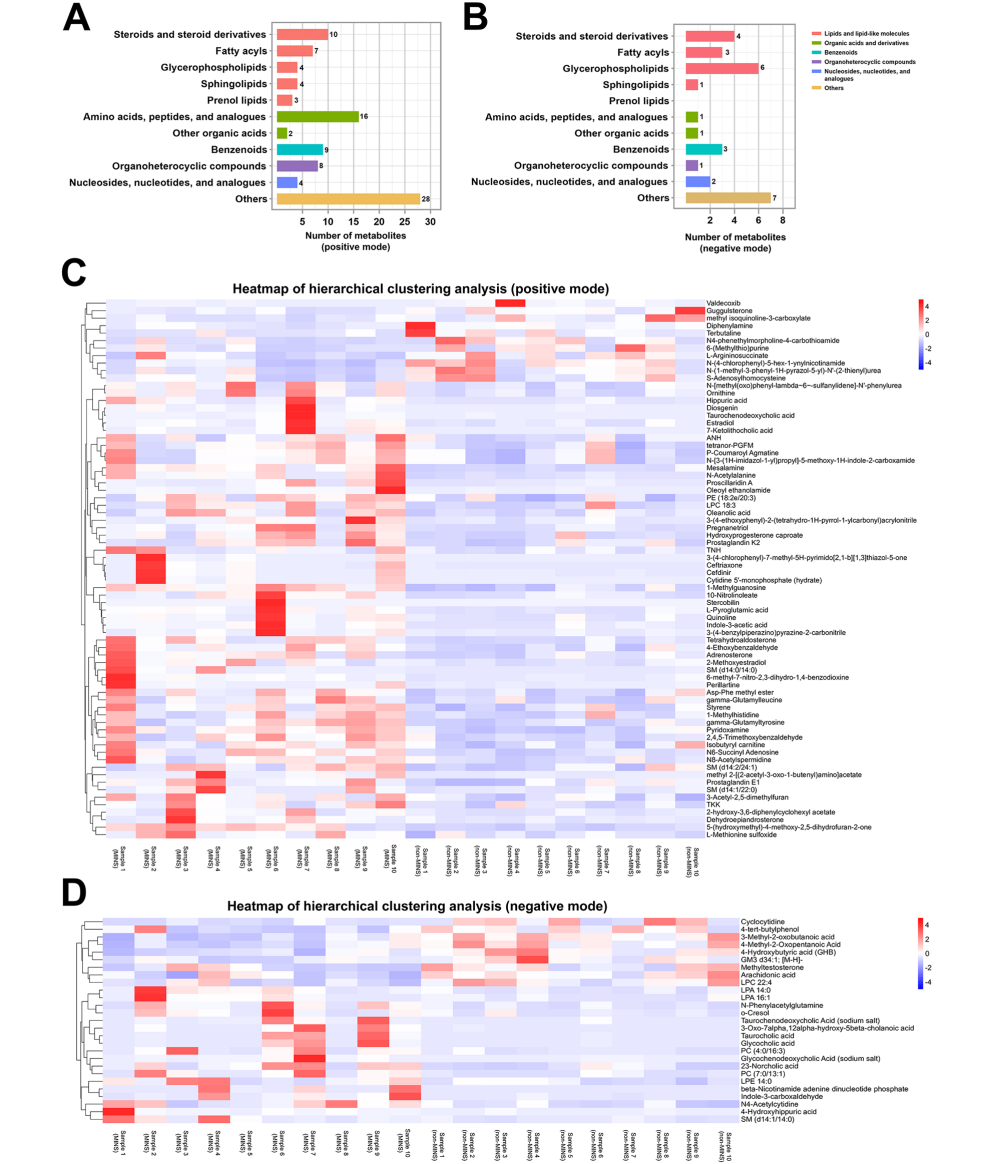
Classification and analysis of differential metabolites in MINS and non-MINS patients. (**A**) classification of differential metabolites in positive mode; (**B**) classification of differential metabolites in negative mode. The X axis represents the number of metabolites within each class, and the Y axis represents the entries for metabolite classification. The term “Others” denotes the remaining categories in the classification information. (**C**) heatmap of hierarchical clustering analysis in positive mode; (**D**) heatmap of hierarchical clustering analysis in negative mode. The red color signifies a relatively high concentration of metabolites, whereas the blue color signifies a relatively low concentration of metabolites. MINS, myocardial injury after noncardiac surgery

Based on various biological functions as determined by the presence of metabolites, differential compounds were mainly classified as “lipids and lipid-like molecules” in both modes (Fig. 3A, B), of which “steroids and steroid derivatives”, “fatty acyls” and “glycerophospholipids” constituted the majority of these compounds in both modes. To further compare the dissimilarities between the two groups, hierarchical clustering analysis was performed, resulting in the generation of heatmaps depicting 125 metabolites (Fig. 3C, D).

**Fig. 4:**
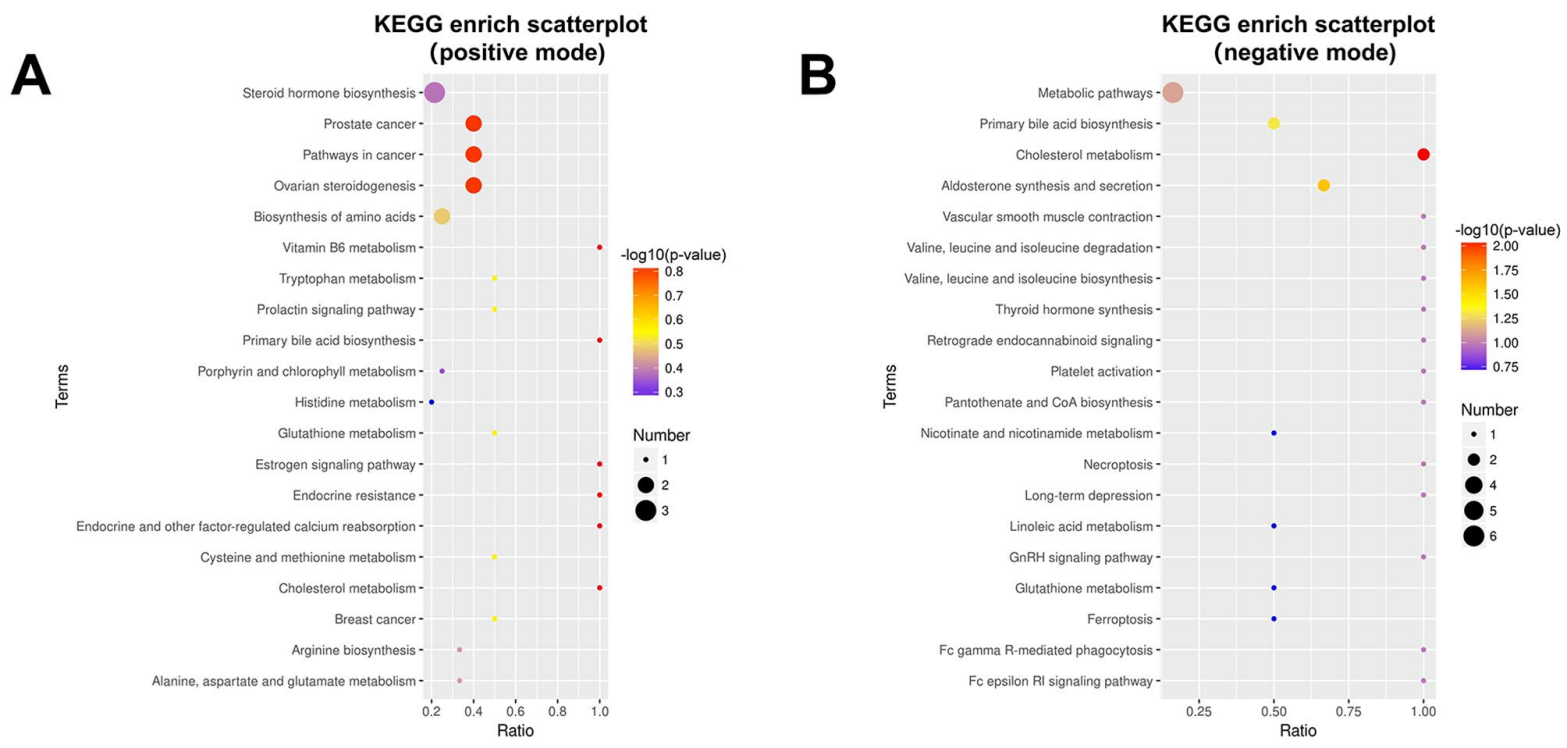
Pathway enrichment analysis of differential metabolites between MINS and non-MINS patients according to the KEGG pathway. (**A**) KEGG enrich scatterplot in positive mode; (**B**) KEGG enrich scatterplot in negative mode. The horizontal coordinate denotes the ratio of differentiated metabolites to the total number of identified metabolites in the corresponding metabolic pathway. The color of the dots corresponds to the *p*-value derived from the hypergeometric test, with smaller values indicating greater significance. Additionally, the size of the dots corresponds to the number of differentiated metabolites within the pathway. KEGG, Kyoto Encyclopedia of Genes and Genomes; MINS, myocardial injury after noncardiac surgery

Subsequently, a KEGG pathway enrichment analysis was conducted, specifically targeting metabolic pathways associated with various biological roles. The resulting KEGG enrich scatterplot (Fig. 4) displayed the top 20 pathways of potential importance. The present analysis has identified significant metabolic signatures, including alterations in cholesterol metabolism, aldosterone synthesis and secretion, primary bile acid biosynthesis, and cysteine and methionine metabolism, distinguishing the MINS patients from the non-MINS patients (*p*-value < 0.05 for hypergeometric test). Notably, the cholesterol metabolism pathway emerged as the most prominent among these metabolic alterations.

### Validation of the potential biomarkers

**Fig. 5:**
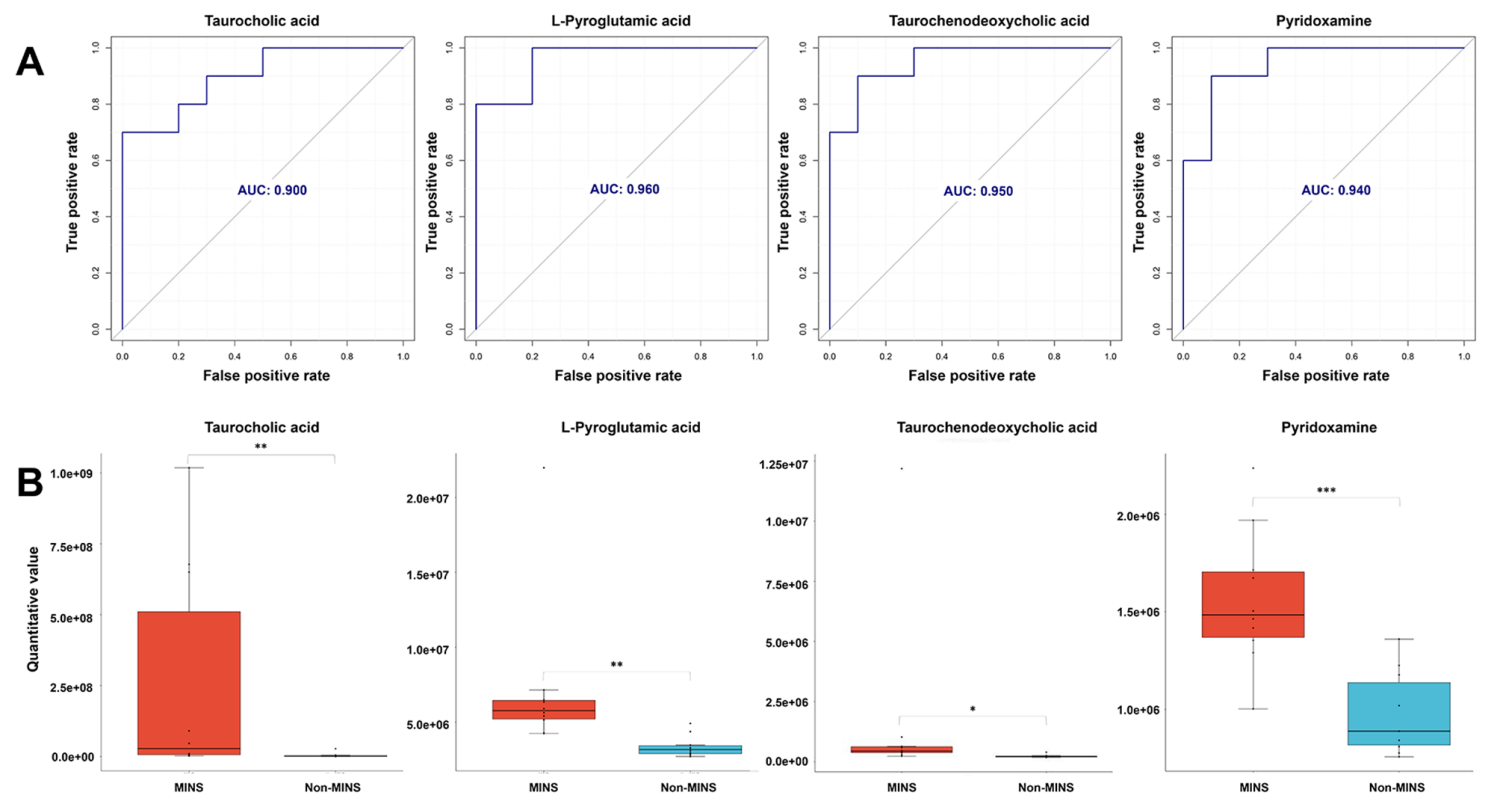
Potential biomarkers of MINS. (**A**) ROC curves of four potential biomarkers with the AUC values above 0.90 and high classification sensitivity (> 75.0%) and specificity (> 75.0%). (**B**) Comparison of the serum levels of four potential biomarkers in MINS and non-MINS patients. AUC, area under curve; MINS, myocardial injury after noncardiac surgery; ROC, receiver operating characteristic. *P < 0.05, **P < 0.01, *** *P* < 0.001

To further assess the potential predictive power of identified biomarkers for MINS, a ROC analysis was conducted on the 124 selected metabolites, showing that 28 metabolites with high AUC above 0.90, which indicated a high level of predictive ability. Among them, four metabolites, including taurocholic acid, L-pyroglutamic acid, taurochenodeoxycholic acid and pyridoxamine, demonstrated classification sensitivity and specificity above 75% (Fig. 5A). Additionally, there was a significant difference of the four metabolites between the MINS and non-MINS patients, as depicted in Fig. 5B.

## Discussion

### Summary of findings

**Fig. 6:**
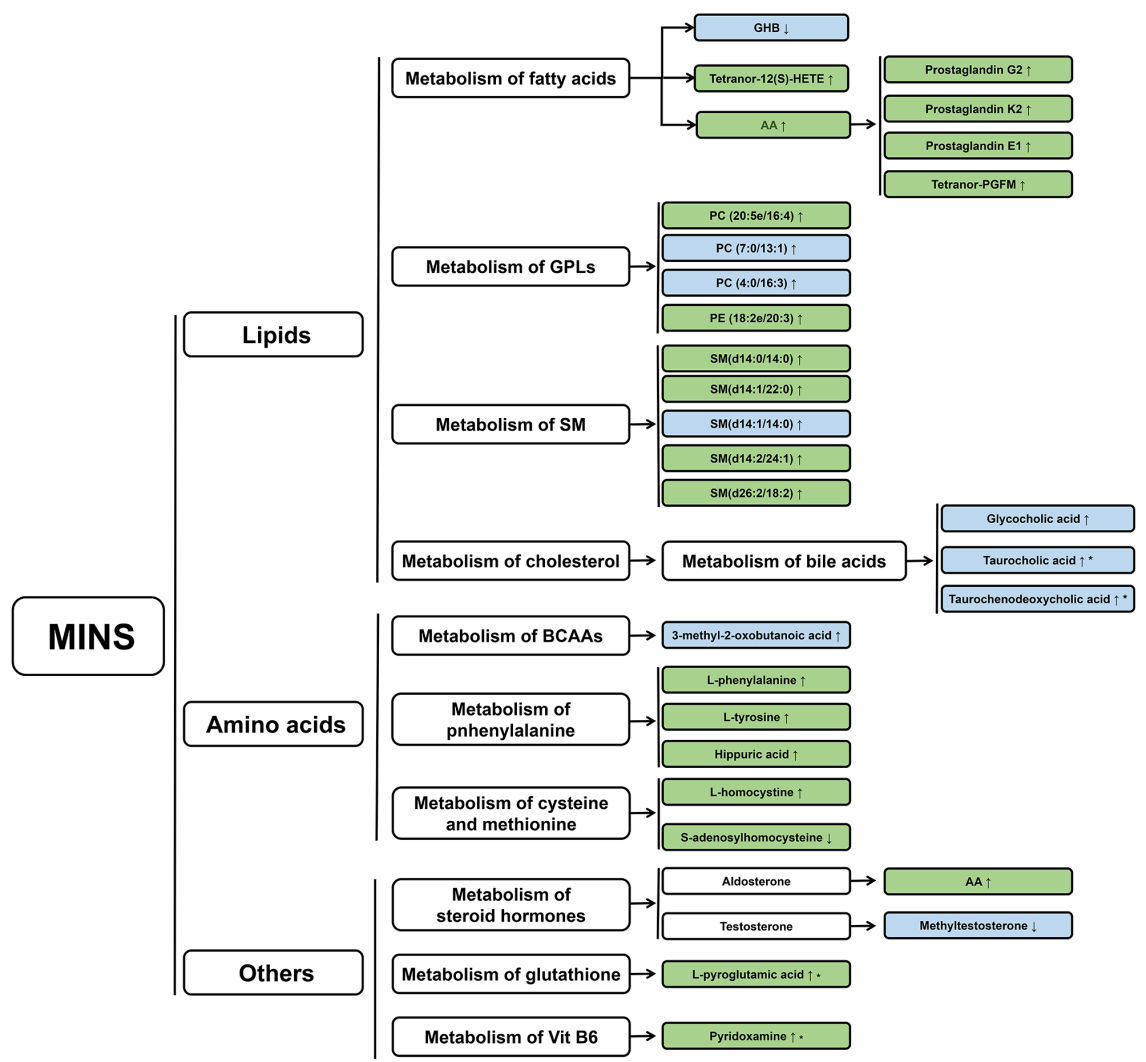
Summary of the altered metabolic processes in MINS patients. Green color represents metabolites in positive mode, while blue color represents metabolites in negative mode. AA, arachidonic acid; BCAAs, branched-chain amino acids; GHB, 4-hydroxybutyric acid; GPLs, glycerophospholipids; HETE, hydroxyeicosatetraenoic acid; MINS, myocardial injury after noncardiac surgery; PGFM, prostaglandin F2alpha metabolite; SM, sphingomyelin; Vit, vitamin. * Potential biomarkers of MINS.

This study determined the metabolite differential expression profile of serum samples obtained from patients with or without MINS using a UHPLC-MS/MS technique. The results indicated significant variations in diverse metabolic pathways between patients with or without MINS. One hundred and twenty-four differential metabolites (95 in positive mode and 29 in negative mode) were detected. As for the important metabolic pathways, four pathways (cholesterol metabolism, aldosterone synthesis and secretion, primary bile acid biosynthesis, and cysteine and methionine metabolism) were revealed by KEGG pathway enrichment analysis. A summary of the altered metabolic processes and related metabolites of this study is presented in Fig. 6. The findings might contribute to the understanding of the pathophysiological occurrence and development process of MINS, which provides cues for discovering diagnostic biomarkers and therapeutic targets of pathophysiological dysfunctions in MINS patients.

### Interpretation of lipids metabolisms in MINS

Lipids play a crucial role in the human body by serving as a primary energy source and forming essential components such as cytomembranes, hormones, and other important biomolecules. Lipid metabolism assumes a critical function, acting as the primary fuel for mediating the oxidative metabolism of the myocardium, thus attributed to driving exacerbation of myocardial injury [16]. Consequently, the exacerbation of MINS can be attributed to the involvement of lipids in this process.

When the myocardial injury occurred, fatty acid uptake and metabolism surged to meet the requirement of the urgent need for energy and metabolic adjustment [17]. In this study, it was observed that Tetranor-12(S)-HETE (hydroxyeicosatetraenoic acid) was markedly upregulated in MINS patients. Previous research has shown that 12(S)-HETE is associated with various cardiovascular biological activities. Ma et al. discovered that the catalytic process of converting arachidonic acid to 12-hydroperoxyeicosatetraenoic acid, facilitated by platelet-type 12-lipoxygenase, was reduced through diverse mechanisms to produce 12(S)-HETE. It has been discovered that small renal arteries are constricted by 12(S)-HETE [18]. Furthermore, 12(S)-HETE significantly impacts angiotensin II-dependent hypertension [19]. Nadler and Satio et al. concluded that angiotensin II-induced aldosterone secretion [20], as well as intracellular calcium transients, was modulated by 12 (S)-HETE [21]. Additionally, research on patients with essential hypertension revealed an elevated level of 12(S)-HETE due to urinary excretion and platelet production [22]. The impact of Tetranor-12(S)-HETE in MINS has remained unclear thus far, but it is a metabolite that warrants further investigation.

Elevated levels of 4-Hydroxybutyric acid (GHB) and arachidonic acid (AA) were observed in the samples obtained from patients with MINS. GHB, an endogenous short-chain fatty acid, is naturally found in the central nervous system and shares structural similarities with γ-aminobutyric acid (GABA), a key inhibitory neurotransmitter [23]. Previous studies have indicated the presence of GHB binding sites in cardiac tissue [24]. Additionally, the enzyme gamma-aminobutyrate transaminase, which plays a crucial role in GABA catabolism, may contribute to cardiac protection by preserving mitochondrial function rather than its GABA catabolic capacity [25]. This suggests potential associations between inhibitory neurotransmitters and myocardial injury.

A non-targeted metabolomics analysis conducted on plasma samples from patients who underwent percutaneous coronary intervention 6 hours post-surgery revealed that the AA metabolism exhibited the most significant alterations among all the metabolites [26]. This discovery emphasizes the significance of investigating AA metabolism in the context of MINS. Numerous studies have indicated that the activation of AA metabolism signals during ischemia-reperfusion directly triggered progressive cardiomyocyte-inflammatory and apoptosis, extensive inflammatory cell infiltration and subsequent cardiac dysfunction [26, 27]. The prostanoids and other biologically active lipids, known as eicosanoids, are synthesized from AA. Zhang et al. conducted studies that suggested prostanoid biosynthesis in cardiomyocytes and the vascular system may serve as a protective mechanism against acute myocardial ischemia-reperfusion injury [28]. Our research identified elevated levels of prostaglandin G2, prostaglandin K2, prostaglandin E1, and tetranor-PGFM (prostaglandin F 2alpha metabolite) in the MI group, which aligns with findings from previous studies.

Glycerophospholipids (GPLs), which are the primary component of cytomembrane and important bioactive lipids such as phosphatidylcholine (PC) and phosphatidylethanolamine (PE), have been found to be associated with clinical outcomes of myocardial injury [29, 30]. In this study, metabolic analyses revealed an upregulation of PC (20:5e/16:4), PC (7:0/13:1), PC (4:0/16:3), and PE (18:2e/20:3) in the MINS group. It was confirmed that pharmacologically increasing bioactive phosphosphingolipids following acute myocardial injury had a beneficial effect on improving physiological function after myocardial infarction [31]. Sphingomyelin (SM), being the principal phosphosphingolipids and a crucial component of cytomembranes, plays a pivotal role in regulating mechanical stability, signaling, and sorting. This research identified upregulated levels of SM (d14:0/14:0), SM (d14:1/22:0), SM (d14:1/14:0), SM (d14:2/24:1), and SM (d26:2/18:2) in the MINS group.

The KEGG analysis revealed that cholesterol metabolism exhibited the most significant alterations. Cholesterol homeostasis is required to maintain proper cellular and systemic functions, and disturbances in cholesterol metabolism have been implicated as a causative factor in cardiovascular disease [32]. Specifically, cholesterol level is a strong risk factor for atherosclerosis and cardiovascular disease. Additionally, cholesterol crystals have been observed predominantly in thrombi originating from plaque rupture of patients who suffered myocardial infarction, although the precise role of cholesterol crystals in thrombi remains to be thoroughly investigated [33]. The primary pathway for cholesterol metabolism involves its conversion into bile acids. Williamson et al. observed that the contraction of cardiomyocytes and the maintenance of intracellular calcium balance were influenced by the primary bile acid taurocholate [34]. Zhang et al. discovered that an abnormal elevation in serum total bile acid levels tends to higher the risk of developing coronary plaques in asymptomatic patients, suggesting that serum bile acid levels could serve as a reliable indicator of acute myocardial injury [35]. These findings suggest a strong association between abnormal bile acid metabolism and the occurrence and progression of myocardial injury [36]. The KEGG analysis revealed that primary bile acid biosynthesis was a pathway that exhibited significant changes, with an observed increase in glycocholic acid, taurocholic acid, and taurochenodeoxycholic acid.

### Interpretation of amino acid metabolisms in MINS

In addition to lipids, amino acids serve as an essential source of energy for cardiovascular adjustments. Specifically, branched-chain amino acids (BCAA), a crucial group of amino acids, act as substrates for oxidation in myocardial tissues [37]. The findings of the studies indicated that the utilization of fatty acids in adult cardiac myocytes could be enhanced through the use of BCAA [38]. Specifically, the level of 3-methyl-2-oxobutanoic acid was upgraded in our experimental results. The observed reduction in 3-methyl-2-oxobutanoic acid levels resulted in impaired BCAA transformation, potentially compromising the utilization of fatty acids, and weakening the myocardial injury protection mechanism [39].

The metabolism of phenylalanine has been identified as a potential profile associated with myocardial injury. The findings of our study revealed that L-phenylalanine, L-tyrosine, and hippuric acid were found to be elevated. Previous research has also demonstrated a correlation between increased levels of serum phenylalanine and coronary failure [40]. Additionally, the process of C4-hydroxylation of phenylalanine into tyrosine, which serves as a precursor for catecholamines that are upregulated in senescence and heart failure, is catalyzed by phenylalanine hydroxylase [41]. Furthermore, another study indicated that alterations in phenylalanine levels might be associated with the incidence of acute rejection in rat heart transplantation [42]. The metabolic pathways of cysteine and methionine have been found to be extensively linked to the occurrence and development of cardiovascular diseases [43], which is consistent with the findings of the KEGG result. The upgraded L-homocystine and downgraded S-adenosylhomocysteine were observed in MINS patients.

### Interpretation of other metabolisms in MINS

The role of hormone metabolism in cardiovascular diseases has been focused over the last several years. The physiological state of steroid hormones, regulated by nongenomic pathways affecting calcium homeostasis, can potentially modify cardiac repolarization [44]. Among all the related hormones, aldosterone and testosterone contribute the most to cardiovascular diseases, especially myocardial injury [45, 46]. The adverse effect of the increase in aldosterone levels has been thoroughly validated in individuals experiencing the acute phase of myocardial injury [45]. Multiple studies have demonstrated that decreased serum testosterone levels can be applied to predicting the mortality of patients with heart failure [46]. Both aldosterone and testosterone appear to play crucial roles in acute myocardial injury. Our findings suggest that aldosterone synthesis and secretion is a significantly enriched pathway in differential metabolites. AA also plays a role in this metabolic pathway. This finding indicated an increase in AA levels, while methyltestosterone levels decreased in patients with MINS.

Additionally, our study observed an elevation in L-pyroglutamic acid and pyridoxamine. Pyroglutamic acid is derived from glutathione degradation, and there is a comprehensive understanding of the involvement of the glutathione cycle in the cellular response to heart function [47]. Pyridoxamine, a natural form of vitamin B6, was identified as a protective factor in cases of cardiac dysfunction resulting from aging or myocardial infarction [48, 49]. These associations suggest potential predictive capabilities for MINS.

### Generalizability

MINS stands as one of the most critical post-surgery complications, significantly linked to postoperative mortality and adverse cardiovascular outcomes. Despite its gravity, the distinctive pathophysiological changes underlying MINS remain elusive, presenting a considerable challenge in timely diagnosis. Our study endeavors to address this gap by shedding light on the metabolomic features of MINS and presenting potential biomarkers. The novel contributions could be summarized as: (1) contextualization of metabolism and biomarkers in MINS: the development and onset of myocardial injury during the perioperative period differ from those in traditional coronary heart disease, influenced by factors such as anesthesia, surgery, and pain. Our focus on the context of MINS allowed us to delineate the specific relevance of the identified molecules in a distinct clinical scenario. By identifying their correlations with MINS, we highlight their potential as specific biomarkers, paving the way for a more tailored diagnostic and prognostic approach for MINS patients; (2) propose a novel strategy for biomarker discovery and potential intervention targets: our study goes beyond the mere identification of biomarkers by integrating bioinformatic analyses subsequent to MS-spectrum analysis. This approach allowed us to unravel intricate relationships between these molecules and additional pathways, revealing potential mechanisms underlying their involvement in MINS. The interconnectedness of these biomarkers with various other molecular pathways sheds new light on the complex molecular landscape of MINS. While these molecules have been implicated in cardiovascular diseases, our study provides a foundation for potential targeted interventions specific to prevent or treat MINS. This holds the promise of yielding a positive impact on patient care and outcomes.

### Limitation

First, the primary constraint of this untargeted metabolomics analysis is the relatively small sample size, although the comprehensive criteria were used for selecting differential metabolites. Therefore, additional investigations involving a larger and more diverse population across multiple time points are necessary to substantiate the reliability of these metabolic findings. Second, the absence of a comparison between pre-surgery (baseline) and post-surgery serum samples restricts the applicability of the results to clinical settings. Collecting preoperative blood samples in future studies is imperative as it may help better grasp the pathophysiology of MINS.

## Conclusion

Lipids metabolism, amino acids metabolism and others exhibited significant differences between the patients with or without MINS. Four specific metabolites may have the potential as biomarkers for MINS. The observed changes in metabolic pathways were primarily associated with cholesterol metabolism, aldosterone synthesis and secretion, primary bile acid biosynthesis and cysteine and methionine metabolism. This study presents valuable information for the metabolomic characteristics of MINS, but future research should involve larger sample sizes and employ targeted metabolomics and other biotechnologies to confirm these findings.

### Electronic supplementary material

Below is the link to the electronic supplementary material.

**Additional Table S1**. Differential metabolites between MINS and non-MINS groups

**Additional Figure S1** Quality control of UHPLC-MS/MS analysis. (A) chromatograms of four QC samples in positive mode; (B) chromatograms of four QC samples in negative mode. (C) Pearson correlation analysis for four QC samples in positive mode; (D) Pearson correlation analysis for four QC samples in negative mode. A higher correlation between the QC samples, as indicated by a closer R^2^ value to 1, suggests improved stability of the overall detection process and enhanced data quality. QC, quality control; UHPLC-MS/MS, ultra-high-performance liquid chromatography-tandem mass spectrometry

## Data Availability

The data and materials used and analysed during the current study are available from the corresponding author on reasonable request.

## Abbreviations

AA: Arachidonic acid
AUC: Area under the curve
BCAA: Branched-chain amino acids
ESI-: Negative electrospray ionization
ESI+: Positive electrospray ionization
GABA: γ-aminobutyric acid
GHB: 4-Hydroxybutyric acid
GPLs: Glycerophospholipids
HETE: Hydroxyeicosatetraenoic acid
KEGG: Kyoto Encyclopedia of Genes and Genomes
MINS: Myocardial injury after noncardiac surgery
PC: Phosphatidylcholine
PCA: Principal component analysis
PE: Phosphatidylethanolamine
PGFM: Prostaglandin F 2alpha metabolite
PLS-DA: Partial least squares discriminant analysis
QC: Quality control
ROC: Receiver operating characteristic
SM: Sphingomyelin
UHPLC-MS/MS: Ultra-high-performance liquid chromatography-tandem mass spectrometry
VIP: Variable importance in the projection

## References

[1] Botto F, Alonso-Coello P, Chan MT, Villar JC, Xavier D, Srinathan S, Guyatt G, Cruz P, Graham M, Wang CY (2014). Myocardial injury after noncardiac surgery: a large, international, prospective cohort study establishing diagnostic criteria, characteristics, predictors, and 30-day outcomes. Anesthesiology.

[2] Devereaux PJ, Biccard BM, Sigamani A, Xavier D, Chan MTV, Srinathan SK, Walsh M, Abraham V, Pearse R, Wang CY (2017). Association of Postoperative High-Sensitivity troponin levels with myocardial Injury and 30-Day mortality among patients undergoing noncardiac surgery. Jama-J Am Med Assoc.

[3] Devereaux PJ, Szczeklik W (2020). Myocardial injury after non-cardiac surgery: diagnosis and management. Eur Heart J.

[4] Devereaux PJ, Biccard BM, Sigamani A, Xavier D, Chan MTV, Srinathan SK, Walsh M, Abraham V, Pearse R, Wang CY (2017). Association of Postoperative High-Sensitivity troponin levels with myocardial Injury and 30-Day mortality among patients undergoing noncardiac surgery. JAMA.

[5] van Waes JA, Nathoe HM, de Graaff JC, Kemperman H, de Borst GJ, Peelen LM, van Klei WA (2013). Cardiac Health after surgery I: myocardial injury after noncardiac surgery and its association with short-term mortality. Circulation.

[6] Iddagoda MT (2021). The role of high-sensitive troponin measurement as a biomarker during the postoperative period for the detection of myocardial injury after non-cardiac surgery. J Perioper Pract.

[7] Devereaux PJ, Xavier D, Pogue J, Guyatt G, Sigamani A, Garutti I, Leslie K, Rao-Melacini P, Chrolavicius S, Yang H (2011). Characteristics and short- term prognosis of Perioperative myocardial infarction in patients undergoing noncardiac surgery a cohort study. Ann Intern Med.

[8] Puelacher C, Lurati Buse G, Seeberger D, Sazgary L, Marbot S, Lampart A, Espinola J, Kindler C, Hammerer A, Seeberger E (2018). Perioperative Myocardial Injury after noncardiac surgery: incidence, mortality, and characterization. Circulation.

[9] May SM, Abbott TEF, Del Arroyo AG, Reyes A, Martir G, Stephens RCM, Brealey D, Cuthbertson BH, Wijeysundera DN, Pearse RM (2020). MicroRNA signatures of perioperative myocardial injury after elective noncardiac surgery: a prospective observational mechanistic cohort study. Br J Anaesth.

[10] Zhao X, Fritsche J, Wang J, Chen J, Rittig K, Schmitt-Kopplin P, Fritsche A, Häring HU, Schleicher ED, Xu G (2010). Metabonomic fingerprints of fasting plasma and spot urine reveal human pre-diabetic metabolic traits. Metabolomics.

[11] Lu W, Jiang Z, Huang J, Bian J, Yu X (2021). Preoperative serum metabolites and potential biomarkers for Perioperative Cognitive decline in Elderly patients. Front Psychiatry.

[12] Kajiura D, Yamanaka-Okumura H, Hirayama A, Tatano H, Endo K, Honma M, Igarashi K, Shoji F, Ikeda S, Yamaguchi N (2019). Perioperative serum and urine metabolome analyses in patients with hepatocellular carcinoma undergoing partial hepatectomy. Nutrition.

[13] Mussap M, Noto A, Fanos V, Van den Anker JN. Emerging Biomarkers and Metabolomics for Assessing Toxic Nephropathy and Acute Kidney Injury (AKI) in Neonatology. *Biomed Res Int* 2014, 2014. 10.1155/2014/602526 PMC4071811 25013791

[14] Huang H, van Dullemen LFA, Akhtar MZ, Faro ML, Yu Z, Valli A, Dona A, Thézénas ML, Charles PD, Fischer R (2018). Proteo-Metabolomics reveals compensation between ischemic and non-injured contralateral kidneys after reperfusion. Sci Rep.

[15] Jammer I, Wickboldt N, Sander M, Smith A, Schultz MJ, Pelosi P, Leva B, Rhodes A, Hoeft A, Walder B (2015). Standards for definitions and use of outcome measures for clinical effectiveness research in perioperative medicine: European Perioperative Clinical Outcome (EPCO) definitions: a statement from the ESA-ESICM joint taskforce on perioperative outcome measures. Eur J Anaesthesiol.

[16] Hall AR, Karwi QG, Kumar S, Dongworth R, Aksentijević D, Altamimi TR, Fridianto KT, Chinda K, Hernandez-Resendiz S, Mahmood MU (2022). Fasting increases susceptibility to acute myocardial ischaemia/reperfusion injury through a sirtuin-3 mediated increase in fatty acid oxidation. Sci Rep.

[17] Ford DA (2002). Alterations in myocardial lipid metabolism during myocardial ischemia and reperfusion. Prog Lipid Res.

[18] Ma YH, Harder DR, Clark JE, Roman RJ (1991). Effects of 12-HETE on isolated dog renal arcuate arteries. Am J Physiol.

[19] Nozawa K, Tuck ML, Golub M, Eggena P, Nadler JL, Stern N (1990). Inhibition of lipoxygenase pathway reduces blood pressure in renovascular hypertensive rats. Am J Physiol.

[20] Nadler JL, Natarajan R, Stern N (1987). Specific action of the lipoxygenase pathway in mediating angiotensin II-induced aldosterone synthesis in isolated adrenal glomerulosa cells. J Clin Invest.

[21] Saito F, Hori MT, Ideguchi Y, Berger M, Golub M, Stern N, Tuck ML (1992). 12-Lipoxygenase products modulate calcium signals in vascular smooth muscle cells. Hypertension.

[22] Gonzalez-Nunez D, Claria J, Rivera F, Poch E (2001). Increased levels of 12(S)-HETE in patients with essential hypertension. Hypertension.

[23] Felmlee MA, Morse BL, Morris ME (2021). Gamma-hydroxybutyric acid: Pharmacokinetics, Pharmacodynamics, and Toxicology. AAPS J.

[24] Maitre M, Klein C, Mensah-Nyagan AG (2016). Mechanisms for the specific properties of gamma-hydroxybutyrate in Brain. Med Res Rev.

[25] Zhang M, Zhong H, Cao T, Huang Y, Ji X, Fan GC, Peng T. Gamma-Aminobutyrate Transaminase protects against lipid overload-triggered Cardiac Injury in mice. Int J Mol Sci 2022, 23(4). 10.3390/ijms23042182 PMC8874535 35216295

[26] Zhang XJ, Liu X, Hu M, Zhao GJ, Sun D, Cheng X, Xiang H, Huang YP, Tian RF, Shen LJ et al. Pharmacological inhibition of arachidonate 12-lipoxygenase ameliorates myocardial ischemia-reperfusion injury in multiple species. *Cell Metab* 2021, 33(10):2059–2075.e2010. 10.1016/j.cmet.2021.08.014 34536344

[27] Olenchock BA, Moslehi J, Baik AH, Davidson SM, Williams J, Gibson WJ, Chakraborty AA, Pierce KA, Miller CM, Hanse EA (2016). EGLN1 inhibition and rerouting of α-Ketoglutarate suffice for remote ischemic protection. Cell.

[28] Zhu L, Zhang Y, Guo Z, Wang M (2020). Cardiovascular Biology of prostanoids and Drug Discovery. Arterioscler Thromb Vasc Biol.

[29] Menger RF, Stutts WL, Anbukumar DS, Bowden JA, Ford DA, Yost RA (2012). MALDI mass spectrometric imaging of cardiac tissue following myocardial infarction in a rat coronary artery ligation model. Anal Chem.

[30] Sousa B, Melo T, Campos A, Moreira AS, Maciel E, Domingues P, Carvalho RP, Rodrigues TR, Girão H, Domingues MR (2016). Alteration in Phospholipidome Profile of myoblast H9c2 cell line in a model of Myocardium Starvation and Ischemia. J Cell Physiol.

[31] Klyachkin YM, Nagareddy PR, Ye S, Wysoczynski M, Asfour A, Gao E, Sunkara M, Brandon JA, Annabathula R, Ponnapureddy R (2015). Pharmacological elevation of circulating Bioactive Phosphosphingolipids enhances myocardial recovery after Acute Infarction. Stem Cells Transl Med.

[32] Luo J, Yang H, Song BL (2020). Mechanisms and regulation of cholesterol homeostasis. Nat Rev Mol Cell Biol.

[33] Alkarithi G, Duval C, Shi Y, Macrae FL, Ariens RAS (2021). Thrombus structural composition in Cardiovascular Disease. Arterioscler Thromb Vasc Biol.

[34] Williamson C, Gorelik J, Eaton BM, Lab M, de Swiet M, Korchev Y (2001). The bile acid taurocholate impairs rat cardiomyocyte function: a proposed mechanism for intra-uterine fetal death in obstetric cholestasis. Clin Sci (Lond).

[35] Zhang BC, Chen JH, Xiang CH, Su MY, Zhang XS, Ma YF (2019). Increased serum bile acid level is associated with high-risk coronary artery plaques in an asymptomatic population detected by coronary computed tomography angiography. J Thorac Dis.

[36] Cao J, Li J, Gu Z, Niu JJ, An GS, Jin QQ, Wang YY, Huang P, Sun JH (2023). Combined metabolomics and machine learning algorithms to explore metabolic biomarkers for diagnosis of acute myocardial ischemia. Int J Legal Med.

[37] Huang Y, Zhou M, Sun H, Wang Y (2011). Branched-chain amino acid metabolism in heart disease: an epiphenomenon or a real culprit?. Cardiovasc Res.

[38] Li Y, Xiong Z, Yan W, Gao E, Cheng H, Wu G, Liu Y, Zhang L, Li C, Wang S (2020). Branched chain amino acids exacerbate myocardial ischemia/reperfusion vulnerability via enhancing GCN2/ATF6/PPAR-alpha pathway-dependent fatty acid oxidation. Theranostics.

[39] Peng YC, Zhao XH, Zeng CF, Xu JX, Qi LN, Li LQ (2022). Integrated omics analysis: the relationship between significantly increased Klebsiella post-hepatectomy and decreased hub-metabolite 3-methyl-2-oxobutanoic acid is associated with induced liver failure. J Gastrointest Oncol.

[40] Tenori L, Hu X, Pantaleo P, Alterini B, Castelli G, Olivotto I, Bertini I, Luchinat C, Gensini GF (2013). Metabolomic fingerprint of heart failure in humans: a nuclear magnetic resonance spectroscopy analysis. Int J Cardiol.

[41] Czibik G, Mezdari Z, Murat Altintas D, Brehat J, Pini M, d’Humieres T, Delmont T, Radu C, Breau M, Liang H (2021). Dysregulated phenylalanine catabolism plays a key role in the trajectory of Cardiac Aging. Circulation.

[42] Tao M, Xiu DR (2013). Metabonomic analysis of rats with acute heart rejection. Transpl Proc.

[43] Lai Q, Yuan GY, Wang H, Liu ZL, Kou JP, Yu BY, Li F (2020). Exploring the protective effects of schizandrol A in acute myocardial ischemia mice by comprehensive metabolomics profiling integrated with molecular mechanism studies. Acta Pharmacol Sin.

[44] Alexandre J, Milliez P, Rouet R, Manrique A, Allouche S, Piccirillo G, Schiariti M, Puddu PE (2015). Aldosterone and testosterone: two steroid hormones structurally related but with opposite electrophysiological properties during myocardial ischemia-reperfusion. Fundam Clin Pharmacol.

[45] Beygui F, Labbé JP, Cayla G, Ennezat PV, Motreff P, Roubille F, Silvain J, Barthélémy O, Delarche N, Van Belle E (2013). Early mineralocorticoid receptor blockade in primary percutaneous coronary intervention for ST-elevation myocardial infarction is associated with a reduction of life-threatening ventricular arrhythmia. Int J Cardiol.

[46] Güder G, Frantz S, Bauersachs J, Allolio B, Ertl G, Angermann CE, Störk S (2010). Low circulating androgens and mortality risk in heart failure. Heart.

[47] Bachhawat AK, Yadav S, Jainarayanan AK, Dubey P (2020). Heart failure and the glutathione cycle: an integrated view. Biochem J.

[48] Wang CH, Wu ET, Wu MS, Tsai MS, Ko YH, Chang RW, Chang CY, Chang KC (2014). Pyridoxamine protects against mechanical defects in cardiac ageing in rats: studies on load dependence of myocardial relaxation. Exp Physiol.

[49] Deluyker D, Ferferieva V, Driesen RB, Verboven M, Lambrichts I, Bito V (2017). Pyridoxamine improves survival and limits cardiac dysfunction after MI. Sci Rep.

