# Supplementary material for: Serum metabolism characteristics of patients with myocardial injury after noncardiac surgery explored by the untargeted metabolomics approach
Source: BMC Cardiovasc Disord. 2024 Feb 3;24:88. doi: 10.1186/s12872-024-03736-y (PMC10838454; doi:10.1186/s12872-024-03736-y)
Supplement: Supplementary file 1 — Additional Table S1. Differential metabolites between MINS and non-MINS groups [file 12872_2024_3736_MOESM1_ESM.docx]

Additional Table S1. Differential metabolites between MINS and non-MINS groups

| **Mode** | **Metabolites** | **RT [min]** | **m/z** | **FC** | **log_2_FC** | **P-value** | **ROC** | **VIP** | **Alteration** |
| --- | --- | --- | --- | --- | --- | --- | --- | --- | --- |
| pos | Stercobilin | 5.595 | 595.3497 | 52.87574 | 5.724534 | 0.040877 | 0.84 | 1.436246 | up |
| pos | Ceftriaxone | 5.314 | 555.0549 | 43.25871 | 5.434919 | 0.000467 | 0.97 | 2.315877 | up |
| pos | Cytidine 5'-monophosphate (hydrate) | 5.135 | 324.0586 | 32.47533 | 5.021272 | 0.009339 | 1 | 1.838735 | up |
| pos | 7-Ketolithocholic acid | 7.376 | 391.2845 | 28.49937 | 4.832858 | 0.011687 | 0.85 | 1.93554 | up |
| pos | Cefdinir | 5.341 | 396.0435 | 26.38317 | 4.721546 | 0.00503 | 0.91 | 2.115974 | up |
| pos | 3-(4-chlorophenyl)-7-methyl-5H-pyrimido[2,1-b] [1,3] thiazol-5-one | 5.137 | 277.0216 | 9.02684 | 3.174221 | 0.047691 | 0.8 | 1.410662 | up |
| pos | methyl 2-[(2-acetyl-3-oxo-1-butenyl) amino] acetate | 5.038 | 200.0921 | 7.802177 | 2.963877 | 0.029737 | 0.84 | 1.399842 | up |
| pos | Diosgenin | 7.484 | 397.3107 | 7.747897 | 2.953805 | 0.008903 | 0.93 | 1.692251 | up |
| pos | Taurochenodeoxycholic acid | 7.518 | 522.2872 | 7.220939 | 2.852186 | 0.0186 | 0.95 | 2.118309 | up |
| pos | SM (d14:0/14:0) | 11.539 | 621.4971 | 6.664568 | 2.736511 | 0.016159 | 0.97 | 2.014112 | up |
| pos | 3-(4-benzylpiperazino) pyrazine-2-carbonitrile | 5.207 | 280.1546 | 6.264699 | 2.647245 | 0.04651 | 0.77 | 1.567517 | up |
| pos | Pregnanetriol | 6.959 | 359.258 | 4.861071 | 2.281274 | 0.000695 | 0.94 | 2.094648 | up |
| pos | 2-hydroxy-3,6-diphenylcyclohexyl acetate | 5.997 | 333.1446 | 4.843042 | 2.275914 | 0.028649 | 0.83 | 1.512205 | up |
| pos | Hippuric acid | 5.366 | 180.0658 | 4.358204 | 2.123734 | 0.003805 | 0.91 | 2.037924 | up |
| pos | Indole-3-acetic acid | 5.765 | 176.0709 | 3.562106 | 1.83273 | 0.000977 | 0.93 | 2.157659 | up |
| pos | SM (d14:1/22:0) | 11.752 | 731.6075 | 3.443468 | 1.783862 | 0.017938 | 0.82 | 1.872996 | up |
| pos | Hydroxyprogesterone caproate | 8.177 | 429.3004 | 3.25418 | 1.702294 | 0.003199 | 0.88 | 2.062678 | up |
| pos | Oleoyl ethanolamide | 7.936 | 344.3161 | 3.207718 | 1.681547 | 0.025314 | 0.93 | 1.871464 | up |
| pos | 5-(hydroxymethyl)-4-methoxy-2,5-dihydrofuran-2-one | 1.199 | 127.0366 | 3.176045 | 1.667231 | 2.7E-05 | 0.96 | 2.915752 | up |
| pos | 10-Nitrolinoleate | 7.481 | 343.2636 | 3.078017 | 1.622001 | 0.001944 | 0.92 | 2.177233 | up |
| pos | Proscillaridin A | 6.859 | 531.296 | 2.93182 | 1.551797 | 0.023578 | 0.82 | 1.743553 | up |
| pos | Perillartine | 5.087 | 166.1229 | 2.920173 | 1.546054 | 0.041861 | 0.9 | 1.759046 | up |
| pos | Quinoline | 5.763 | 130.0654 | 2.914673 | 1.543334 | 0.003944 | 0.86 | 1.916021 | up |
| pos | Tetrahydroaldosterone | 6.751 | 365.2323 | 2.877481 | 1.524807 | 0.000426 | 0.92 | 2.297515 | up |
| pos | 2-Methoxyestradiol | 5.349 | 303.1915 | 2.829832 | 1.500717 | 0.034239 | 0.79 | 1.675624 | up |
| pos | Estradiol | 7.401 | 273.1852 | 2.70042 | 1.433184 | 0.006155 | 0.88 | 1.889169 | up |
| pos | N6-Succinyl Adenosine | 4.971 | 384.1149 | 2.48644 | 1.314082 | 0.000111 | 0.96 | 2.302018 | up |
| pos | 6-methyl-7-nitro-2,3-dihydro-1,4-benzodioxine | 4.951 | 196.0606 | 2.268984 | 1.182046 | 0.021074 | 0.94 | 1.734497 | up |
| pos | LPC 18:3 | 8.487 | 518.322 | 2.262093 | 1.177658 | 0.005081 | 0.84 | 1.785575 | up |
| pos | 2,4,5-Trimethoxybenzaldehyde | 6.1 | 197.0812 | 2.25424 | 1.172641 | 0.005879 | 0.84 | 1.736417 | up |
| pos | 3-(4-ethoxyphenyl)-2-(tetrahydro-1H-pyrrol-1-ylcarbonyl) acrylonitrile | 7.234 | 271.1447 | 2.235718 | 1.160738 | 0.020989 | 0.84 | 1.597939 | up |
| pos | N-Acetylalanine | 1.25 | 132.0658 | 2.233505 | 1.159309 | 0.001275 | 0.98 | 1.994707 | up |
| pos | L-Pyroglutamic acid | 5.366 | 147.0765 | 2.149644 | 1.104098 | 0.001539 | 0.96 | 1.982164 | up |
| pos | gamma-Glutamyltyrosine | 4.888 | 311.1238 | 2.111828 | 1.078493 | 0.007224 | 0.83 | 1.502347 | up |
| pos | Prostaglandin K2 | 7.829 | 333.2039 | 2.095555 | 1.067332 | 0.026085 | 0.78 | 1.626939 | up |
| pos | N-[methyl(oxo)phenyl-lambda~6~-sulfanylidene]-N'-phenylurea | 1.142 | 313.0359 | 2.052982 | 1.037721 | 0.007254 | 0.82 | 1.796559 | up |
| pos | TNH | 6.307 | 393.1522 | 2.046837 | 1.033397 | 0.045555 | 0.73 | 1.226418 | up |
| pos | Ornithine | 1.09 | 133.0973 | 1.980097 | 0.985571 | 0.007691 | 0.81 | 1.741669 | up |
| pos | tetranor-PGFM | 5.79 | 313.1621 | 1.919978 | 0.94109 | 0.007195 | 0.87 | 1.17562 | up |
| pos | N8-Acetylspermidine | 1.231 | 188.1758 | 1.893624 | 0.92115 | 0.015335 | 0.83 | 1.56972 | up |
| pos | Dehydroepiandrosterone | 7.121 | 289.2165 | 1.892592 | 0.920364 | 0.034102 | 0.85 | 1.659536 | up |
| pos | Adrenosterone | 6.821 | 301.1792 | 1.841642 | 0.880993 | 0.005015 | 0.83 | 2.150676 | up |
| pos | ANH | 6.172 | 341.1574 | 1.83999 | 0.879698 | 0.041723 | 0.78 | 1.403093 | up |
| pos | Mesalamine | 1.23 | 154.0475 | 1.829971 | 0.871821 | 0.000116 | 0.99 | 2.12512 | up |
| pos | Asp-Phe methyl ester | 5.336 | 295.1294 | 1.821877 | 0.865426 | 0.005045 | 0.87 | 1.894703 | up |
| pos | Prostaglandin E1 | 9.425 | 337.2352 | 1.783192 | 0.834462 | 0.012649 | 0.76 | 1.854583 | up |
| pos | 1-Methylguanosine | 4.772 | 298.1149 | 1.736114 | 0.795861 | 2.96E-05 | 0.97 | 2.414134 | up |
| pos | SM (d14:2/24:1) | 11.26 | 755.6062 | 1.718928 | 0.781509 | 0.033776 | 0.78 | 1.416927 | up |
| pos | N-[3-(1H-imidazol-1-yl) propyl]-5-methoxy-1H-indole-2-carboxamide | 5.668 | 299.147 | 1.664125 | 0.734764 | 0.042486 | 0.78 | 1.071589 | up |
| pos | L-Methionine sulfoxide | 1.791 | 166.0535 | 1.663677 | 0.734375 | 0.010263 | 0.85 | 1.251428 | up |
| pos | 4-Ethoxybenzaldehyde | 6.275 | 151.0755 | 1.647155 | 0.719976 | 0.002636 | 0.9 | 1.891388 | up |
| pos | Pyridoxamine | 4.818 | 169.0973 | 1.603965 | 0.681643 | 0.000134 | 0.94 | 1.869243 | up |
| pos | P-Coumaroyl Agmatine | 5.674 | 277.1648 | 1.597547 | 0.675859 | 0.042149 | 0.78 | 1.107194 | up |
| pos | TKK | 6.524 | 376.2596 | 1.597358 | 0.675688 | 0.016833 | 0.84 | 1.72855 | up |
| pos | PE (18:2e/20:3) | 10.887 | 752.5744 | 1.594681 | 0.673268 | 0.011415 | 0.83 | 1.618072 | up |
| pos | Isobutyryl carnitine | 5.541 | 232.1547 | 1.572505 | 0.653065 | 0.025525 | 0.77 | 1.350068 | up |
| pos | 3-Acetyl-2,5-dimethylfuran | 6.178 | 139.0756 | 1.56522 | 0.646366 | 0.008225 | 0.8 | 1.401088 | up |
| pos | gamma-Glutamylleucine | 5.33 | 244.1182 | 1.544817 | 0.627436 | 0.022496 | 0.81 | 1.386275 | up |
| pos | Styrene | 5.805 | 105.0705 | 1.530426 | 0.613933 | 0.004768 | 0.84 | 1.567451 | up |
| pos | Oleanolic acid | 8.922 | 439.3571 | 1.517558 | 0.601751 | 0.013346 | 0.8 | 1.066965 | up |
| pos | 1-Methylhistidine | 1.218 | 170.0926 | 1.505174 | 0.58993 | 0.029885 | 0.78 | 1.306678 | up |
| pos | DL-α-Aminocaprylic acid | 5.577 | 160.1336 | 1.497383 | 0.582443 | 0.030144 | 0.76 | 1.148433 | up |
| pos | SM (d26:2/18:2) | 10.597 | 837.6734 | 1.493435 | 0.578635 | 0.029643 | 0.82 | 1.213325 | up |
| pos | Lysopc 16:1 | 7.322 | 494.3264 | 1.459761 | 0.545732 | 0.0109 | 0.83 | 1.733607 | up |
| pos | 3-(4-fluorophenoxy)-1-(1,4-thiazinan-4-yl) propan-1-one | 1.182 | 253.0684 | 1.43715 | 0.523211 | 0.012029 | 0.91 | 1.991025 | up |
| pos | N-Benzylformamide | 2.241 | 136.0759 | 1.420634 | 0.506535 | 0.045921 | 0.78 | 1.321386 | up |
| pos | L-Phenylalanine | 4.856 | 166.0863 | 1.405486 | 0.491069 | 0.016421 | 0.79 | 1.656393 | up |
| pos | Sphingosine (d18:1) | 9.149 | 322.2743 | 1.395669 | 0.480956 | 0.028416 | 0.76 | 1.197674 | up |
| pos | SDMA | 1.309 | 203.1503 | 1.393955 | 0.479184 | 0.034365 | 0.8 | 1.475923 | up |
| pos | PC (20:5e/16:4) | 8.021 | 758.5126 | 1.388823 | 0.473863 | 0.025509 | 0.83 | 1.232925 | up |
| pos | Prostaglandin G2 | 6.276 | 369.2274 | 1.383108 | 0.467914 | 0.022467 | 0.89 | 1.474678 | up |
| pos | DL-Citrulline | 1.26 | 176.1031 | 1.37472 | 0.459138 | 0.014735 | 0.83 | 1.623924 | up |
| pos | 4-hydroxy-5,8-dimethylquinoline-3-carboxylic acid | 5.144 | 218.0814 | 1.372927 | 0.457255 | 0.033278 | 0.74 | 1.100046 | up |
| pos | L-Tyrosine | 2.237 | 182.0814 | 1.368681 | 0.452786 | 0.040768 | 0.74 | 1.280111 | up |
| pos | 2-Hydroxycinnamic acid | 2.243 | 165.0548 | 1.350711 | 0.433719 | 0.035943 | 0.77 | 1.313707 | up |
| pos | 3-(3,4-dihydroxyphenyl)propanoic acid | 1.189 | 165.0525 | 1.341494 | 0.423841 | 0.004364 | 0.87 | 1.312915 | up |
| pos | 5-Hydroxytryptophan | 5.144 | 221.0922 | 1.339217 | 0.42139 | 0.041281 | 0.75 | 1.272113 | up |
| pos | 2-Hydroxyphenylalanine | 1.857 | 182.0815 | 1.336988 | 0.418986 | 0.037292 | 0.73 | 1.251622 | up |
| pos | Tetranor-12(S)-HETE | 7.339 | 249.1853 | 1.331993 | 0.413587 | 0.013143 | 0.81 | 1.556939 | up |
| pos | L-Threonine | 1.255 | 120.0659 | 1.315966 | 0.396123 | 0.043155 | 0.77 | 1.18561 | up |
| pos | 7-methyl-3-nitroimidazo[1,2-a]pyridine | 1.183 | 200.0408 | 1.315079 | 0.39515 | 0.000961 | 0.92 | 2.334483 | up |
| pos | L-Homocystine | 1.169 | 269.0632 | 1.31493 | 0.394986 | 0.004377 | 0.84 | 1.848836 | up |
| pos | Sinapinic acid | 1.18 | 247.0579 | 1.220322 | 0.287261 | 0.023109 | 0.82 | 1.599518 | up |
| pos | 3-Succinoylpyridine | 1.18 | 163.0367 | 1.203915 | 0.267734 | 0.010907 | 0.79 | 1.313828 | up |
| pos | L-Argininosuccinate | 8.846 | 291.1298 | 0.578097 | -0.79062 | 0.031199 | 0.78 | 1.787208 | down |
| pos | 6-(Methylthio)purine | 5.829 | 207.0318 | 0.570192 | -0.81048 | 0.03654 | 0.78 | 1.387522 | down |
| pos | methyl isoquinoline-3-carboxylate | 5.036 | 170.0563 | 0.557593 | -0.84271 | 0.028552 | 0.79 | 1.353159 | down |
| pos | Diphenylamine | 6.955 | 170.0966 | 0.540656 | -0.88722 | 0.035385 | 0.85 | 1.875757 | down |
| pos | N4-phenethylmorpholine-4-carbothioamide | 6.387 | 289.0736 | 0.523108 | -0.93482 | 0.027938 | 0.77 | 1.588164 | down |
| pos | N-(1-methyl-3-phenyl-1H-pyrazol-5-yl)-N'-(2-thienyl) urea | 6.692 | 299.0944 | 0.453111 | -1.14206 | 0.019255 | 0.77 | 1.736416 | down |
| pos | S-Adenosylhomocysteine | 6.79 | 385.1312 | 0.429779 | -1.21833 | 0.044952 | 0.77 | 1.533964 | down |
| pos | Terbutaline | 4.81 | 226.144 | 0.382092 | -1.38801 | 0.044845 | 0.77 | 1.323834 | down |
| pos | N-(4-chlorophenyl)-5-hex-1-ynylnicotinamide | 6.967 | 313.1098 | 0.353927 | -1.49848 | 0.002451 | 0.9 | 2.156632 | down |
| pos | Guggulsterone | 6.001 | 313.2162 | 0.341964 | -1.54808 | 0.026517 | 0.81 | 1.717268 | down |
| pos | Valdecoxib | 6.026 | 315.0801 | 0.0679 | -3.88043 | 0.022811 | 0.81 | 1.487623 | down |
| neg | 3-Methyl-2-oxobutanoic acid | 4.735 | 115.0403 | 0.642933 | -0.63726 | 0.000386 | 0.93 | 2.515607 | down |
| neg | 4-Hydroxybutyric acid (GHB) | 3.463 | 103.0402 | 0.556828 | -0.8447 | 0.002301 | 0.9 | 2.206936 | down |
| neg | 4-Methyl-2-Oxopentanoic Acid | 5.589 | 129.0557 | 0.616203 | -0.69852 | 0.002319 | 0.86 | 1.991252 | down |
| neg | Taurocholic acid | 8.477 | 514.2844 | 59.165 | 5.886672 | 0.002601 | 0.9 | 2.090815 | up |
| neg | 23-Norcholic acid | 6.956 | 393.2651 | 3.760932 | 1.91109 | 0.003259 | 0.86 | 1.946818 | up |
| neg | PC (7:0/13:1) | 7.795 | 608.3576 | 4.589303 | 2.198275 | 0.005103 | 0.86 | 1.854144 | up |
| neg | D- (-)-Fructose | 1.261 | 215.0328 | 0.688806 | -0.53783 | 0.007897 | 0.84 | 1.86243 | down |
| neg | LPA 14:0 | 8.589 | 381.2048 | 2.652082 | 1.407125 | 0.008235 | 0.85 | 1.814035 | up |
| neg | N-Phenylacetylglutamine | 5.371 | 108.0454 | 3.482251 | 1.80002 | 0.008389 | 0.83 | 1.68296 | up |
| neg | N4-Acetylcytidine | 4.829 | 284.089 | 1.940129 | 0.956153 | 0.011365 | 0.83 | 1.576428 | up |
| neg | 4-Hydroxyhippuric acid | 4.938 | 194.0456 | 8.857288 | 3.146865 | 0.012003 | 0.87 | 1.882303 | up |
| neg | SM (d14:1/14:0) | 11.212 | 663.4728 | 5.121437 | 2.356549 | 0.012476 | 0.79 | 1.874326 | up |
| neg | Taurochenodeoxycholic Acid (sodium salt) | 10.444 | 498.2897 | 10.1981 | 3.350229 | 0.012639 | 0.84 | 1.685836 | up |
| neg | Glycocholic acid | 6.937 | 464.3015 | 53.61199 | 5.744484 | 0.013536 | 0.83 | 1.881401 | up |
| neg | GM3 d34:1; [M-H]- | 11.84 | 1151.705 | 0.460133 | -1.11988 | 0.016925 | 0.77 | 1.7335 | down |
| neg | Arachidonic acid | 9.825 | 303.2329 | 0.645033 | -0.63256 | 0.018527 | 0.8 | 1.607535 | down |
| neg | Cyclocytidine | 9.427 | 224.0686 | 0.306554 | -1.70579 | 0.019316 | 0.78 | 1.684852 | down |
| neg | beta-Nicotinamide adenine dinucleotide phosphate | 5.327 | 742.0665 | 179.5511 | 7.48825 | 0.019521 | 0.82 | 1.650785 | up |
| neg | Glycochenodeoxycholic Acid (sodium salt) | 6.86 | 448.3073 | 23.7701 | 4.571076 | 0.021952 | 0.85 | 2.009871 | up |
| neg | LPC 22:4 | 9.509 | 616.3625 | 0.616292 | -0.69831 | 0.022811 | 0.78 | 1.473466 | down |
| neg | 3-Oxo-7alpha,12alpha-hydroxy-5beta-cholanoic acid | 7.028 | 405.2648 | 12.22659 | 3.611951 | 0.022906 | 0.8 | 2.104314 | up |
| neg | 4-tert-butylphenol | 6.389 | 149.0972 | 0.611881 | -0.70868 | 0.025441 | 0.8 | 1.255044 | down |
| neg | Indole-3-carboxaldehyde | 5.348 | 144.0455 | 3.461564 | 1.791424 | 0.027008 | 0.83 | 1.428578 | up |
| neg | PC (4:0/16:3) | 7.516 | 604.326 | 2.929019 | 1.550418 | 0.031926 | 0.77 | 1.728466 | up |
| neg | LPA 16:1 | 9.511 | 407.2203 | 2.71138 | 1.439027 | 0.033992 | 0.79 | 1.549921 | up |
| neg | Methyltestosterone | 9.72 | 301.2172 | 0.495202 | -1.01391 | 0.034702 | 0.74 | 1.70992 | down |
| neg | o-Cresol | 5.656 | 107.0504 | 3.777872 | 1.917574 | 0.036455 | 0.76 | 1.456499 | up |
| neg | LPE 14:0 | 8.46 | 424.247 | 2.020871 | 1.014977 | 0.042524 | 0.72 | 1.729188 | up |
| neg | 2,3-dinor Prostaglandin E1 | 7.515 | 307.1916 | 1.356542 | 0.439934 | 0.049538 | 0.77 | 1.401886 | up |

FC, fold change; MINS, myocardial injury after noncardiac surgery; m/z, mass charge ratio; ROC, receiver operating characteristic; RT, retention time; VIP, variable importance in the projection.
