# Supplementary figures and images for: Serum metabolism characteristics of patients with myocardial injury after noncardiac surgery explored by the untargeted metabolomics approach
Source: BMC Cardiovasc Disord. 2024 Feb 3;24:88. doi: 10.1186/s12872-024-03736-y (PMC10838454; doi:10.1186/s12872-024-03736-y)

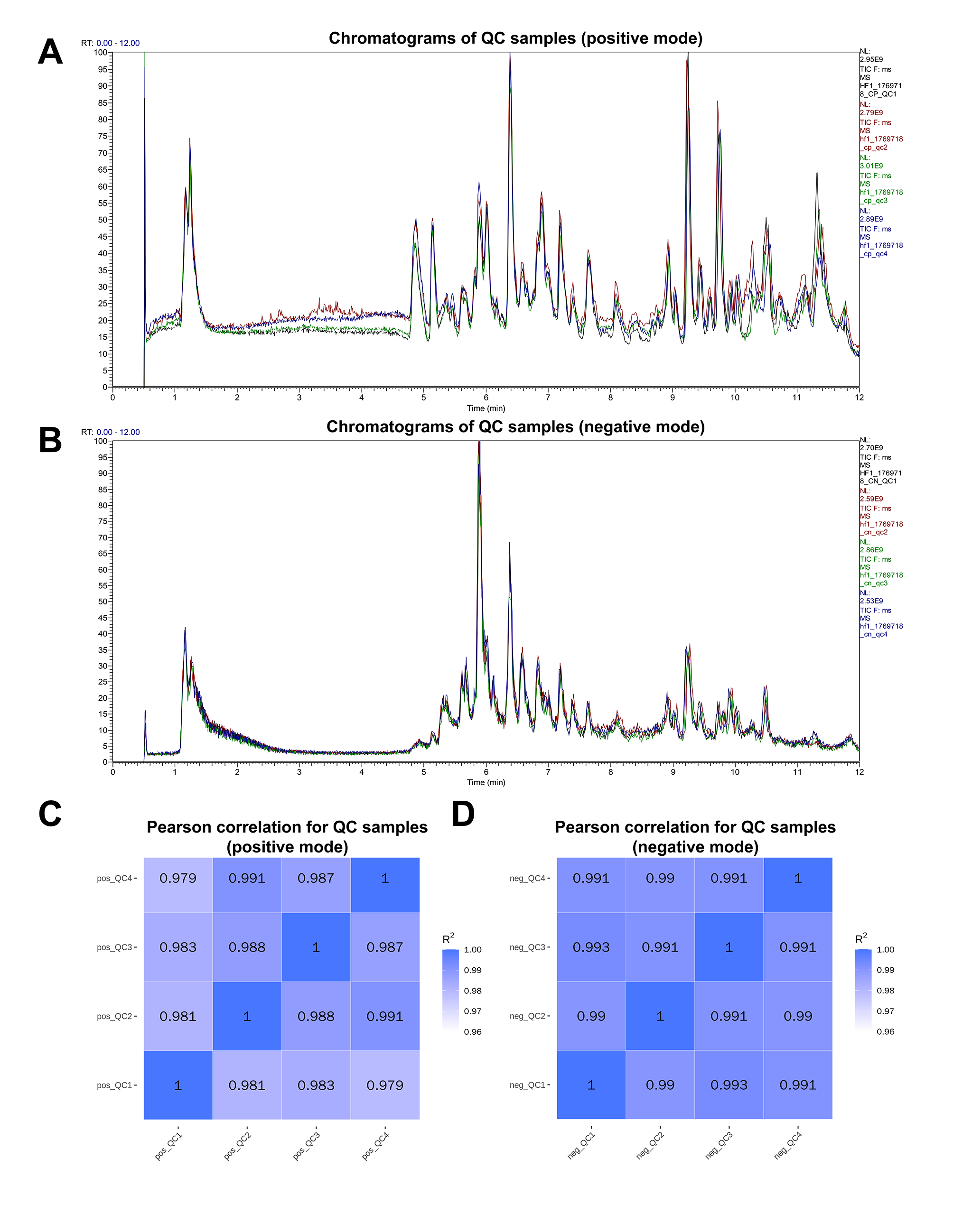

Supplement: Supplementary file 2 — Additional Figure S1 Quality control of UHPLC-MS/MS analysis. (A) chromatograms of four QC samples in positive mode; (B) chromatograms of four QC samples in negative mode. (C) Pearson correlation analysis for four QC samples in positive mode; (D) Pearson correlation analysis for four QC samples in negative mode. A higher correlation between the QC samples, as indicated by a closer R2 value to 1, suggests improved stability of the overall detection process and enhanced data quality. QC, quality control; UHPLC-MS/MS, ultra-high-performance liquid chromatography-tandem mass spectrometry [file 12872_2024_3736_MOESM2_ESM.tif]
